# Editorial: The interplay of plant biotic and abiotic stresses: mechanisms and management

**DOI:** 10.3389/fpls.2024.1518678

**Published:** 2024-11-22

**Authors:** Esther Menéndez, Silvio Tundo

**Affiliations:** ^1^ Departamento de Microbiología y Genética, Universidad de Salamanca, Salamanca, Spain; ^2^ Institute for Agribiotechnology Research (CIALE), Villamayor, Salamanca, Spain; ^3^ Unidad Asociada Grupo de Interacción Planta-Microorganismo, Universidad de Salamanca-IRNASA-CSIC, Salamanca, Spain; ^4^ Department of Land, Environment, Agriculture, and Forestry (TESAF), University of Padova, Legnaro, Italy; ^5^ Department of Agronomy, Food, Natural Resources, Animals and Environment (DAFNAE), University of Padova, Legnaro, Italy

**Keywords:** WRKY transcription factors, receptor-like kinases, comparative transcriptomics, induced systemic resistance (ISR), drought, flooding, *Spilocaea oleaginea*, *Cytospora plurivora*

The challenges posed to modern agriculture such as climate change, depleting groundwater resources, and increased global population (Ivanovich et al., 2023; Khatri et al., 2024), underscore the urgent need for innovative and sustainable strategies to mitigate plant stress. Plants face a multitude of biotic and abiotic stressors, which can severely affect their growth, development, and productivity. Despite these challenges, plants have evolved complex defence mechanisms that are tightly regulated at various molecular levels (Zandalinas et al., 2021; Zandalinas and Mittler, 2022). However, the ways in which biotic and abiotic stresses together influence plant responses at molecular level remain poorly understood.

This Research Topic includes comprehensive studies exploring the interplay between biotic and abiotic stresses, with a particular focus on how abiotic stress can either enhance or reduce plant susceptibility to pathogens and pests. The Research Topic includes a balanced mix of original research and review articles, with an emphasis on molecular and multi-omic approaches. The studies underscore the critical role of transcription factors and kinases in regulating integrative plant responses, utilizing a variety of omics and molecular techniques. One of the published research articles adopts a more applied perspective, emphasizing the importance of *in situ* field measurements for practical implementation.

Within the first type of studies, Zhang et al. summarized studies positioning WRKY transcription factor subfamilies as reference candidates to overcome different stresses such as cold, salinity, nutrient deficiency. Their role against biotic stresses have been proven, i.e., modulating plant immunological responses against pathogen invasion, including effector-triggered immunity (ETI) and pathogen-associated molecular pattern (PAMP)-triggered immunity (PTI). Pathogen infection or elicitors induce the expression of WRKY transcription factors across a broad range of plant species. WRKY transcription factors are homeostatic regulators of ROS and of several flavonoid biosynthesis genes. The deep knowledge of WRKY transcription factors regulation will favour the development of testable hypotheses for fine-tuning the flavonoids accumulation promoting crops resilience to biotic and abiotic stresses.

Liu et al. reviewed the role of receptor-like kinases (RLKs) in several processes crucial for plants, such as regulating cellular activities and signalling cascades elicited in response to biotic and abiotic stresses. Using a comprehensive genome-wide screening approach, Li et al. analysed the genomic architecture, genetic lineages, chromosomal localization, gene replication events, preserved motifs, and expression patterns across different coconut tissues. Specifically, the authors analysed *R2R3-MYB* genes as essential factors involved in anthocyanins, carotenoids and flavonoid biosynthesis in a variety of plants, including coconut. These genes also serve as genetic markers, as they are regulated in response to a range of biotic and abiotic stresses, helping to protect plants from pathogens and mitigate cold and other stress conditions.

Using comparative transcriptomics, Marchese et al. investigated the mechanisms underlying the resistance of olive cultivars to *Spilocaea oleaginea*, the fungal pathogen responsible for Peacock’s eye or leaf spot disease. The disease is highly prevalent in the Mediterranean region, with its impact largely influenced by climatic conditions. The authors conducted RNA-seq analysis on leaf tissues from two olive cultivars: one with low susceptibility (Koroneiki) and one highly susceptible (Nocellara del Belice) to identify putative biomarkers linked to the infection. They detected a thaumatin-like gene over-expressed in both cultivars. Moreover, genes such as *DMR6-LIKE OXYGENASE 2-like*, *MLO*, *DOWNY MILDEW RESISTANCE 6-like*, and alpha carbonic anhydrase were up-regulated in the high susceptible cultivar, being target genes considered as susceptibility factors. Remarkably, many transcription factors involved in Induced Systemic Resistance (ISR) and abiotic stress response were found to be uniquely expressed in low susceptible cultivar and none in the high susceptible cultivar. All of those findings point out to the development of genetic biomarkers for screening the olive germplasm for vulnerability against stress responses.

In the category of applied field studies, Miller et al. evaluated thirteen peach cultivars under drought and high pH conditions, for susceptibility to the pathogen *Cytospora plurivora*, responsible for producing *Cytospora* canker in peach orchards. High pH and drought stress enhanced peach susceptibility to the pathogen. The authors proposed that cultural practices might be directed to prevent drought stress and ensure the quality of water distributed within orchards, avoiding water with high pH and/or concentrations of soluble salts. This study highlights the importance of incorporating abiotic stress factors into the assessment of plant responses to pathogens.

Gui et al. reviewed transcriptomic, proteomic, and metabolomic studies of model plants and major crops subjected to flooding to elucidate potential mechanisms and adaptive strategies of plants to this stress. The authors highlighted that several processes typically involved in plant response to biotic stress such as cell wall remodelling, ROS regulation, protein phosphorylation, flavonoid biosynthesis and sugar metabolism are significantly altered in plants under flooding conditions.

In conclusion, targeted research on plant regulatory mechanisms for both biotic and abiotic stresses is essential for understanding how plants respond to combined stress factors. Omics represents an essential technological resource to decipher achieve this goal. The ability to pinpoint molecular targets involved in plant response to both stress categories and incorporate them into breeding programs will be a critical step in developing broad-spectrum stress-tolerant crops.
